# Carboxy terminal collagen crosslinks as a prognostic risk factor for fall-related fractures in individuals with established spinal cord injury

**DOI:** 10.1038/s41393-019-0322-0

**Published:** 2019-07-15

**Authors:** Vivien Jørgensen, Hanne Bjørg Slettahjell, Kirsti Skavberg Roaldsen, Emil Kostovski

**Affiliations:** 1 Department of Research, Sunnaas Rehabilitation Hospital, University of Oslo, Oslo, Norway; 2 Division of Physiotherapy, Department of Neurobiology, Care Sciences and Society, Karolinska Institutet, Stockholm, Sweden; 3 Faculty of Health Sciences, Department of Physiotherapy, Oslo Metropolitan University, Oslo, Norway

**Keywords:** Physiology, Biomarkers

## Abstract

**Study design:**

Prospective cohort study.

**Objective:**

To study associations between specific bone turnover markers and fall-related fractures in individuals with spinal cord injury (SCI).

**Setting:**

Rehabilitation Hospital.

**Methods:**

Carboxy terminal collagen crosslinks (CTX), type-1 procollagen N-terminal (P1NP), albumin-corrected calcium (Ca^2+^), parathyroid hormone (PTH) and vitamin D were examined in a cohort of 106 participants with SCI at least 1 year post injury. The participants were followed for 1 year monitoring fall-related fractures.

**Results:**

In total, 29 out of 106 reported having experienced a fall-related fracture post-injury at baseline, and 5 out of 100 had experienced a fall-related bone fracture during the 1 year follow-up. Our main findings were that high levels of serum CTX increased the odds of being in the fracture group, and that 25-hydroxy vitamin D (25 OHD) levels, Ca^2+^, PTH or P1NP were not associated with being in the fracture group.

**Conclusions:**

We here present an association between high-CTX plasma levels at baseline and fall-related fractures reported during a 1-year follow-up among individuals with established SCI. We recommend studies with larger SCI populations before further clinical implications can be drawn.

## Introduction

Paralysed muscles and diminished vertical load on bones below the neurological level of injury result in extensive bone loss early after a spinal cord injury (SCI). This results in an accelerated condition of osteopenia or osteoporosis and is a frequent cause of bone fractures among individuals with established SCI [1–4]. Loss of bone strength the first months and years after SCI exceeds loss of bone after prolonged bed rest, and in some individuals the loss equals that of a lifetime in able-bodied [5, 6]. Hormonal changes, low weight load and reduced muscle activity are proposed mediators, in addition to age, gender, family history and lifestyle factors such as smoking, alcohol consumption and vitamin D status. Prevention strategies to reduce fracture incidence are important to avoid additional restrictions in physical function after SCI.

The modified fragility assessment score (FRAX) has been introduces as a tool to pinpoint individuals with a high risk of acquiring fractures [7]. However, the FRAX score is not specific to the SCI population. Sufficient vitamin D levels in combination with dietary calcium are important physiological factors that maintain bone mineral density [8, 9]. Sub-optimal levels of vitamin D causes bone mineral loss through secondary hyperpara-thyroidism, and low serum levels of 25-hydroxy vitamin D (25 OHD) have been shown to predict hip fracture in the elderly [10]. Individuals with SCI have an increased risk of vitamin D deficiency due to reduced sun exposure, bed rest and low physical activity [11]. Carboxy terminal collagen (CTX), a crosslink peptide sequence of type I collagen, is a biomarker of bone turnover. Its serum levels are proportional to osteoclastic activity at the time the blood sample is drawn [12, 13]. We have previously reported CTX serum concentration among individuals with a post-acute SCI to be 10 times higher than among able-bodied [2], and studies have reported high CTX values as a predictor of fractures in able-bodied [14–17]. There is no data on whether high CTX levels predict fractures in individuals with SCI, or evidence concerning optimal vitamin D status after an SCI, and their association with fractures after SCI. Such data could be of value in clinical practice to identify those at risk. In this study, the aim was to determine if specific bone turnover markers can be used to predict fall-related fractures.

## Methods

### Inclusion, setting and data collection procedures

This 1 year, prospective study was part of a multi-centre study: the Spinal Cords Injury Prevention of Falls (SCIP Falls) Study, conducted at Sunnaas Rehabilitation Hospital, Norway, and Rehab Station Stockholm/Spinalis, Sweden, in collaboration with Karolinska Institutet, Sweden [18–20]. Participants were consecutively recruited at regular follow-ups between February 2013 and February 2014 in the catchment areas of South-East Norway, and participants in the present study constitutes the total Norwegian cohort of the SCIP falls study. Inclusion criteria were: traumatic SCI; at least 1 year post injury; minimum age of 18 years; mastering the Norwegian language and understanding the aim of the project. Individuals that were included were classified according to the International Standards for Neurological Classification of SCI [21]. Exclusion criteria were: motor complete injuries above C5, injuries below L5 level or injuries classified as American Spinal Injury Association (ASIA) Impairment Scale (AIS) E (normal sensory and motor functions) [21]. The outcomes of interest were fall-related fractures and their relation to bone-specific parameters and vitamin D status by 25 OHD levels. A fall was defined as “an unexpected event in which the participants come to rest on the ground, floor, or lower level” [18, 22]. Falls were monitored for 1 year by sending a text message via an online short message service (SMS -Track ApS, Esbjerg, Denmark) every other week. If a participant’s SMS reply was ‘yes’, a structured telephone interview was conducted within a week, focusing on number of falls, and why, how, when and where the fall happened, as well as possible injuries. All participants were telephoned for 4, 8 and 12 months after baseline to maintain compliance and collect fall data. See previous published data for a detailed description [18, 19]. Antecubital vein blood samples were collected from the participants once, in the morning after overnight fast at the beginning of the follow-up period. Serum samples were aliquoted, quick-frozen and stored at −20 °C before further analyses. CTX crosslinks (lower detection limit 0.07 μg/l, CV 10%) and type-1 procollagen N-terminal (P1NP, lower detection limit 5 μg/l, CV 2–4%) were assayed by an electrochemoluminescence immunoassay using a Roche E 170 module by Roche Diagnostics (F. Hoffmann-La Roche Ltd, Basel, Switzerland). Parathyroid hormone (PTH, lower detection limit 0.5 pmol/l, CV 6–7%) was measured with a non-competitive immunoluminometric assay using an Immulite 2000 by Siemens Healthcare Diagnostics (Erlangen, Germany). Bone-specific alkaline phosphatase (b-ALP, lower detection limit 9.5 µg/l, CV 10–13%) was measured after immune extraction with a kit from QUIDEL Corp. (San Diego, CA, USA). We measured 25 OHD (lower detection limit 13 nmol/l, CV 13–16%) with a radioimmunoassay from DiaSorin (Stillwater, MN, USA). Phosphate, CRP and albumin-corrected calcium (Ca^2+^) were measured with routine, immunometric assays using the Vitros 250 Chemistry System by Ortho Clinical Diagnostics (Johnson & Johnson, Auckland, New Zealand).

### Statistical analyses

The statistical analyses were performed with SPSS version 25 (Chicago, IL). Descriptive statistics included median, ranges, means and standard deviations (SD), together with simple tests including Fishers-exact and *χ*^2^ tests to compare categorical variables and *t-*tests to compare continuous variables. The participants were divided in a fracture group, i.e., those who had experienced one or more bone fracture(s) during the 1 year follow-up due to a fall, and a non-fracture group. We performed simple correlation tests and a binary logistic regression analysis with fracture group versus non-fracture group as the dependent variable, to study factors that might be associated with having experienced one or more fractures at baseline and after fall during the 1-year follow-up. We excluded variables that explained a similar relationship. Age, gender, body mass index (BMI) and completeness of injury (categorised as wheelchair user or ambulatory, mode of mobility was dichotomised as at least 75% wheelchair use or ambulatory [18, 20]) were included in the logistic regression with the use of the Enter method. Up to five more variables, were added with the backward likelihood ratio (LR) method: CTX, vitamin D plasma levels, previous fractures, time after injury (y) and alcohol consumption (yes/no). Alcohol consumption was defined as yes if you drink more than 9 (women) and 14 (men) standardised units of alcohol per week (WHO guidelines). If a *p*-value ≤ 0.1, the variable were included. Values are reported as odds ratios (ORs) with intercept (*B*) with standard error and 95% confidence intervals (CIs). We considered *p*-values < 0.05 to indicate statistical significance.

## Results

### Characteristics of the study participants

The total study population demographics (*n* = 106) are described in Table 1. Gender ratio was 1:5 for female:male. The age of the participants at the time of the interview ranged from 19 to 83 years (mean (SD); 49 (16) years), and time since injury was between 1 and 51 years; 15 (12) years). BMI ranged from 14 to 40 kg/m^2^; 25 (5) kg/m^2^). Neurological level ranged from to C1 to L3. Sixty-two participants were defined as wheelchair users and 44 as ambulatory.

**Table 1:** Baseline characteristics and neurological classification of the participants according to ISNCSCI (*N* = 106)

Male, gender, *n* (%)	86 (81)
Age, mean (SD)	49 (16)
BMI, mean (SD)	25 (5)
Years from injury, median (range)	11 (1–51)
Number of prospective falls, median (range)	1 (0–11)
*Nevrological level,* n *(%)*	
Cervical	54 (51)
Thoracal 1–6	16 (15)
Thoracal 7–12	28 (26)
Lumbar	8 (8)
*Americal Impairment Scale (AIS),* n *(%)*	
AIS A,B or C C4 and above	9 (8)
AIS A,B or C C5-C8	14 (13)
AIS A,B or C Th1-S5	41 (39)
AIS D any level	42 (40)

*BMI* body mass index, *ISNCSCI* International Standard Neurological Classification of Spinal Cord Injury

### Fractures and biomarkers of bone turnover

**Fig. 1:**
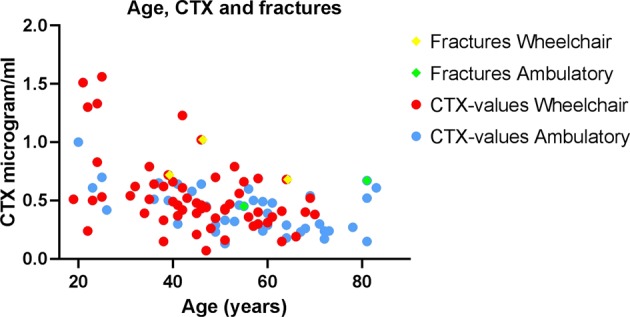
Fracture distribution across CTX blood levels and age (years). Ambulatory individuals are marked with blue and wheelchair dependent are marked with red dots. Fractures among wheelchair dependent are marked with yellow squares and ambulatory with green squares. WC wheelchair, Amb ambulatory, CTX carboxy terminal collagen

**Fig. 2:**
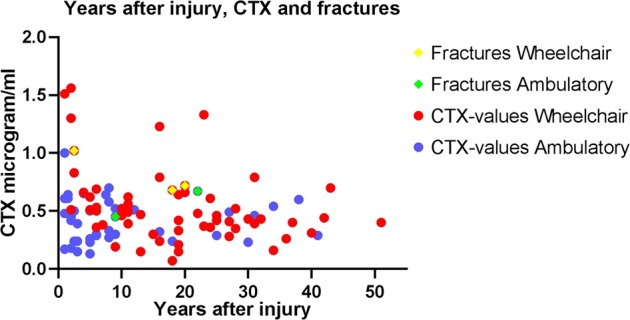
Fracture distribution across CTX blood levels and time after injury (years). Ambulatory individuals are marked with blue dots and wheelchair dependent are marked with red dots. Fractures among wheelchair dependent are marked with yellow squares and ambulatory with green squares. WC wheelchair, Amb ambulatory, CTX carboxy terminal collagen

Table 2 shows biomarkers of bone turnover assessed in our cohort. All parameters were within the reference range of the able-bodied population. Twenty-nine participants reported at baseline to have experienced a fall-related fracture post-injury. The distribution of fractures was most frequently around the knee or proximal femur. Five individuals experienced six fractures related to falling during the 1-year follow-up, including one femur-, two tibia/fibula-, one costa-, one wrist- and two hand-bone-fracture(s), see Table 3. Blood markers were not retrieved from six of the participants; however, no fractures were reported from these. Figure 1 show the fracture distribution across CTX blood levels and age, Fig. 2 shows fracture distribution across CTX blood levels and years after injury. CTX plasma levels correlated with years from injury in the wheelchair dependent participants (*r* = −0,35, *p* = 0.006), but not the ambulatory (*r* = −0.002, *p* = 0.898). Table 4a shows the results of the association between different variables on fracture group and non-fracture group during the 1-year follow-up. Our model explained from 9 to 26% of the relationship between the two groups (fracture and non-fracture). The logistic regression analysis revealed that every unit increase in CTX increased the risk of being in the prospective fracture group (*p* = 0.021), however, the CTX confidence interval (CI) was very wide (95% CI 2.0–4769.6). Table 4b shows the results of the association between different variables on fracture group and non-fracture group at baseline. Our model explained from 23 to 24% of the relationship between the two groups (fracture and non-fracture). The logistic regression analysis revealed that increased time after injury (y) increased the risk of being in the fracture group (*p* < 0.001) at baseline. We found no association between P1NP, Ca^2+^, PTH and acquired fractures during the 1-year follow-up. A larger proportion of our study population had sub-optimal levels of 25 OHD (≤50 nmol/l), 39 and 62% for ambulatory and wheelchair users respectively. The mean values were lower with greater variation during the winter months for both groups. During the summer months, mean values of the wheelchair users were still sub-optimal, whereas the ambulatory participants had satisfactory levels of vitamin D during these months (≥50 nmol/l). We found no correlation between sub-optimal 25 OHD levels (cut-off level calculated for 30, 50 and 70 nmol/l) and fall-induced fractures.

**Table 2:** Biological markers of bone turnover (*n* = 100)

	Men (*n* = 86)	Women (*n* = 20)
PTH (pmol/l)	4.4 (1.9)	4.1 (1.9)
Ca2 + (mmol/l)	1.2 (0.04)	1.2 (0.04)
b-ALP (µg/l)	32 (15)	31 (10)
CTX_I (µg/l)	0.5 (0.3)	0.4 (0.1)
PINP (µg/l)	55 (33)	54 (19)
Vit_D3 (nmol/l)	47 (20)	53 (20)

Numbers are presented as mean (SD), *p*-value = 0.05

*CTX* carboxy terminal collagen, *P1NP* type-1 procollagen N-terminal, *Ca2 +* albumin-corrected calcium, *PTH* parathyroid hormone and vitamin D3

**Table 3:** Descriptives and bone markers of patients who reported fractures during 1 year follow-up (*n* = 5)

Gender	Age	Years post-injury	Injury level/ AIS score	Wheelchair user/ ambulatory %	Bone markers						Fracture type	Previous fractures
	(years)				BMI (kg/m^2^)	PTH (pmol/l)	Calcium (mmol/l)	CTX (μg/l)	Vit D (nmol/l)	P1NP (μg/l)		
Male	39	20	C4/A	100/0	19.9	4.8	1.2	0.72	37	87	Prox, tibia	No
Male	46	2.5	T10/A	100/0	25.9	3.2	1.2	1.02	16	177	Prox, tibia and fibula	No
Male	64	18	T1/A	100/0	19.5	4.2	1.21	0.68	45	104	Femur	Femur
Female	55	9	T5/A	25/75	33.5	5.7	1.19	0.45	79	96	Rib and thumb at 2 occasions	No
Female	81	22	C1/D	0/100	21.5	2.7	1.18	0.69	70	80	Carpal bone	Wrist

Numbers are absolute

*AIS* American Injury Scale, *BMI* Body Mass Index, *PTH* parathyroid hormone, *CTX* Carboxy terminal collagen crosslinks, *Vit D* 25(OH)vitamin D; *P1NP* type-1 procollagen N-terminal

**Table 4a:** Prospective fall-related fractures-logistic regression model

Variables associated with fall-related fractures	*B*	S.E.	*P*-value	OR	Confidence intervals	
				Lower	Upper	
Age (years)	0.1	0.0	0.07	1.1	1.0	1.2
Gender (male/female)	−1.4	1.4	0.32	0.2	0.0	3.9
BMI (kg/m*m)	0.0	0.1	0.89	1.0	0.8	1.2
WC or ambulatory	1.1	1.5	0.48	3.0	0.1	61.6
CTX (microgram/l)	4.6	2.0	0.02	97.9	2.0	4769.6

Variables included in a multivariate regression model. Mode of mobility was dichotomised as at least 75% wheelchair use or ambulatory. Model summary: Cox & Snell *R*^2^: 0.089

*B* intercept, *OR* odds ratio, *CTX* carboxy terminal collagen

*p-value < 0.05

**Table 4b:** Fractures after injury-logistic regression model

Variables associated with fall-related fractures	*B*	S.E.	Sig.	OR	Confidence interval	
					Lower	Upper
Gender (man/woman)	−0.79	0.64	0.22	0.46	0.13	1.59
BMI (kg/m*m)	−0.05	0.05	0.31	0.95	0.86	1.05
WC or ambulator	0.33	0.63	0.60	1.39	0.40	4.80
Age (years)	0.02	0.02	0.31	1.02	0.98	1.06
Time from injury (years)	0.09	0.02	0.00	1.09	1.05	1.15

Variables included in a multivariate regression model. Mode of mobility was dichotomised as at least 75% wheelchair use or ambulatory. Model summary: Cox & Snell *R*^2^:0.23

*B* intercept, *SE* standard error, *OR* odds ratio

*p-value < 0.05

## Discussion

We here describe fall-related fractures over a period of 1 year in 106 individuals with SCI and the association to bone biomarkers in serum. In total, 29 out of 106 reported to have had a fall-related fracture at baseline, and 5 out of 106 individuals had experienced a fall-related bone fracture during the 1-year follow-up. Our main findings were that high levels of serum CTX increased the odds of being in the fracture group and that low 25 OHD levels (cut off 30, 50 or 75 nmol/l) were not associated with being in the fracture group. CTX serum levels as well as all other markers of bone turnover were within reference range of the able-bodied reference population. According to Marx et al. [12]. CTX plasma levels in the able-bodied population usually range up to 450 pg/ml. In the osteoporosis population, values are commonly 400 pg/mL to 550 pg/mL in patients not taking bisphosphonates. All our participants with fractures had ≤450 pg/ml. Increased CTX levels increased the risk of being in the fracture group more than variables such as age, ambulation and gender (see Table 4a). However, the CI was wide, most probably due to low total outcome. Finding no associations may merely reflect small numbers of fractures, and thus our results should be interpreted with caution. Even so, based on our study and studies from able-bodied where serum CTX has been found to be associated with fracture risk [12, 16, 23], we believe the association between high-CTX plasma levels and increased fracture risk also to be true for individuals with established SCI. We acknowledge the need for further studies in larger SCI populations. We believe that increased CTX plasma levels should be discussed as a fracture risk factor in addition to other risk factors commonly used in risk assessments scores and also as a monitor of osteoporosis treatment among individuals with SCI. Studies from the able-bodied population have identified CTX in blood to be the reference markers of bone turnover when monitoring osteoporosis treatment [23], and there are today outpatient clinics that monitor osteoporosis treatment with CTX levels also among individuals with SCI. We did not find any correlation between sub-optimal 25 OHD levels and fall-induced fractures. Interestingly, a recent meta-analysis in able-bodied community-dwelling older adults found no association with fracture incidence and supplementation of calcium, vitamin D, or combined calcium and vitamin D [24].

Twenty-seven percentage of our study group reported fractures at baseline with a 5% fracture rate during the 1-year follow-up. Higher numbers of years since injury were associated with a higher risk of fracture post injury at baseline (see Table 4b), which is similar to previous reports [6, 25–30]. Two out of five individuals in the prospective fall-related fracture group had experienced a previous fracture (see Table 3).This is similar to other studies which show that the patients with SCI who suffer one fracture, are then more likely to experience fracture again [31].

### Strengths and limitations

The strength of this study is the prospective design combined with blood sampling prior to the fracture event. There are relatively few fractures in our study population during the 1-year follow-up, making our statistical calculations prone to misinterpretation. Also, the inclusion of ambulatory subjects could reduce the prognostic value of the dataset for those with complete injuries, and the focus on fall-related fractures may include fractures other than fragility fractures associated with sub-lesional osteoporosis. Furthermore, we did not register fractures not related to falls, such as fatigue and stress fractures. Still, our close follow-up would most likely reveal any non-fall-related fracture. We did analyse the blood samples continuously, and we have no objective measures of osteoporosis of our study participants.

## Conclusions

We here present an association between high-CTX plasma levels at baseline and fall-related fractures reported during a 1-year follow-up among individuals with established SCI. We recommend studies with larger SCI populations before further clinical implications can be drawn.

## Data Availability

The datasets generated and/or analysed during the current study are available from the corresponding author on reasonable request.
